# Breast cancer risk assessment across the risk continuum: genetic and nongenetic risk factors contributing to differential model performance

**DOI:** 10.1186/bcr3352

**Published:** 2012-11-05

**Authors:** Anne S Quante, Alice S Whittemore, Tom Shriver, Konstantin Strauch, Mary B Terry

**Affiliations:** 1 Department of Epidemiology, Columbia University, 722 West 168th Street 724A, New York, NY 10032, USA; 2 Institute of Medical Informatics, Biometry and Epidemiology, Chair of Genetic Epidemiology, Ludwig-Maximilians-Universität, 81377 Munich, Germany; 3 Institute of Genetic Epidemiology, Helmholtz Zentrum München - German Research Center for Environmental Health, 85764 Neuherberg, Germany; 4 Department of Health Research and Policy, Stanford University School of Medicine, Stanford, CA 94305, USA; 5 Herbert Irving Comprehensive Cancer Center, Columbia University Medical Center, 701 West 168th Street, New York, NY 10032, USA

## Abstract

**Introduction:**

Clinicians use different breast cancer risk models for patients considered at average and above-average risk, based largely on their family histories and genetic factors. We used longitudinal cohort data from women whose breast cancer risks span the full spectrum to determine the genetic and nongenetic covariates that differentiate the performance of two commonly used models that include nongenetic factors - BCRAT, also called Gail model, generally used for patients with average risk and IBIS, also called Tyrer Cuzick model, generally used for patients with above-average risk.

**Methods:**

We evaluated the performance of the BCRAT and IBIS models as currently applied in clinical settings for 10-year absolute risk of breast cancer, using prospective data from 1,857 women over a mean follow-up length of 8.1 years, of whom 83 developed cancer. This cohort spans the continuum of breast cancer risk, with some subjects at lower than average population risk. Therefore, the wide variation in individual risk makes it an interesting population to examine model performance across subgroups of women. For model calibration, we divided the cohort into quartiles of model-assigned risk and compared differences between assigned and observed risks using the Hosmer-Lemeshow (HL) chi-squared statistic. For model discrimination, we computed the area under the receiver operator curve (AUC) and the case risk percentiles (CRPs).

**Results:**

The 10-year risks assigned by BCRAT and IBIS differed (range of difference 0.001 to 79.5). The mean BCRAT- and IBIS-assigned risks of 3.18% and 5.49%, respectively, were lower than the cohort's 10-year cumulative probability of developing breast cancer (6.25%; 95% confidence interval (CI) = 5.0 to 7.8%). Agreement between assigned and observed risks was better for IBIS (HL X_4_^2 ^= 7.2, *P *value 0.13) than BCRAT (HL X_4_^2 ^= 22.0, *P *value <0.001). The IBIS model also showed better discrimination (AUC = 69.5%, CI = 63.8% to 75.2%) than did the BCRAT model (AUC = 63.2%, CI = 57.6% to 68.9%). In almost all covariate-specific subgroups, BCRAT mean risks were significantly lower than the observed risks, while IBIS risks showed generally good agreement with observed risks, even in the subgroups of women considered at average risk (for example, no family history of breast cancer, *BRCA1/2 *mutation negative).

**Conclusions:**

Models developed using extended family history and genetic data, such as the IBIS model, also perform well in women considered at average risk (for example, no family history of breast cancer, *BRCA1/2 *mutation negative). Extending such models to include additional nongenetic information may improve performance in women across the breast cancer risk continuum.

## Introduction

Accurate assessment of a woman's absolute breast cancer risk is needed in clinical management decisions about mammographic screening, risk-reducing surgeries and other preventive interventions. In the United States, annual screening mammography and magnetic resonance imaging (MRI) beginning at age 30 years are recommended for women with a lifetime risk of 20% or greater [1]. In addition, the Breast Cancer Risk Assessment Tool (BCRAT, also called the Gail model) is used to determine whether a woman meets the minimum risk threshold of a five-year risk of at least 1.67% for considering tamoxifen for chemoprevention [1,2].

Several statistical models have been developed for assigning absolute risk of developing breast cancer [3-6]. Some models are based solely on family history, such as the Claus model [7], some are based on family history, *BRCA1/2 *carrier status, and polygenes such as the BOADICEA model [5], whereas others incorporate nongenetic risk factors, such as the BCRAT model [3,8-10] and the International Breast Cancer Intervention Study model (IBIS, also called the Tyrer Cuzick model) [6]. The BCRAT model is the most frequently used breast cancer risk assessment tool in the U.S. [11]. This model includes current age, age at menarche, age at first live birth, number of previous biopsies, history of atypical hyperplasia, race/ethnicity and number of affected first-degree female relatives. However, it does not include information on *BRCA1/2 *mutation status or extended family history (meaning breast cancers in male relatives, number and breast cancer status/ovarian cancer status of second-degree relatives, and age of onset of all affected relatives). In contrast, the IBIS model includes extended family history, *BRCA1/2 *genetic status with nongenetic risk factors such as age, age at menarche, parity, age at first live birth, age at menopause, history of hormone replacement therapy use, history of hyperplasia/atypical hyperplasia, history of lobular carcinoma *in situ*, height and body mass index (BMI).

**Figure 1:**
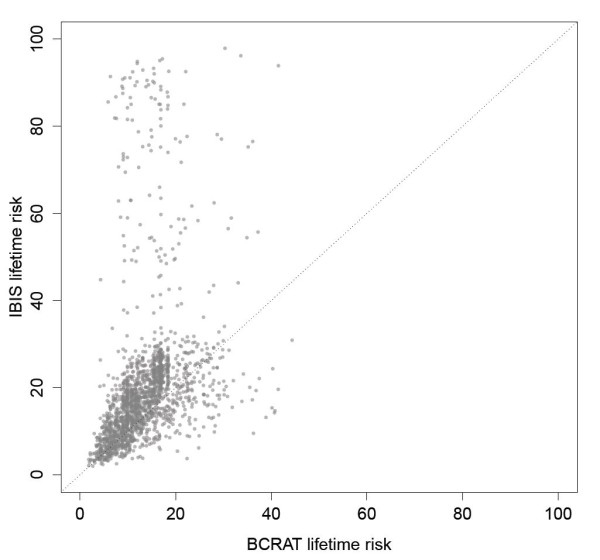
**Scatterplot of BCRAT and IBIS lifetime risks**. The horizontal and vertical coordinates of points give the 1857 subjects' lifetime risks as assigned by BCRAT and IBIS, respectively. The two sets of assigned risks are only weakly correlated (Pearson's correlation coefficient r = 0.34).

The BCRAT model has been evaluated in several large cohorts [9,12-14] and has been found well calibrated for women at average risk, its discriminatory ability is more moderate (median c-statistic of 0.59, (reviewed in [15]) [16-18]. It is well known that the short-term and lifetime breast cancer risks assigned to a woman by BCRAT and IBIS vary considerably. For example, Figure 1 shows weak correlation (r = 0.34) between the lifetime risks assigned by BCRAT and IBIS to the 1,857 participants in the current study. The BCRAT model tends to assign lower risks than the IBIS model to women with a strong family history of breast cancer than does the IBIS model [19]. Indeed, the BCRAT model has not been recommended for these women, nor for women aged under 35 years at risk assessment or with a personal history of lobular (LCIS) or ductile carcinoma *in situ *(DCIS) [20]. Consequently clinicians typically use models like BCRAT for women deemed at average risk and models like IBIS for women whose family history and genetic information indicate above-average risk.

Here, we compare these two models as they are applied clinically because they capture both nongenetic and family history data, and they are commonly used in the clinic. A single model applicable to all women would be useful, particularly in view of the large differences apparent in Figure 1 (range of absolute difference 0.001 to 79.5). The BCRAT and IBIS models are widely used in the United States and currently they are the only two models that incorporate both genetic and nongenetic factors. Comparison of these models using prospective cohort data has been very limited; for example, one study of 1,933 women, of whom 52 developed cancer during an average follow up of 5.27 years compared both the BCRAT and IBIS models to each other [16]. In the current cohort study, we compare the calibration and discrimination of BCRAT and IBIS within subgroups of women determined by the levels of their assigned risks and by genetic and nongenetic covariates. Our objective was to compare model performance in subgroups of women typically thought to be of average risk (for example, women without a strong family history or a *BRCA1/2 *mutation) versus subgroups typically classified as above-average risk.

## Materials and methods

### Study population

The New York site of the Breast Cancer Family Registry (BCFR) has recruited and followed 4,991 participants (4,064 women and 927 men) from 1,322 families since 1995 (for details see [21-26]. Eligible subjects fulfilled the following criteria: two or more relatives with a personal history of breast or ovarian cancer; a woman diagnosed with breast or ovarian cancer at a young age (<45 years), a women with a personal history of both breast and ovarian cancer; an affected male with breast cancer in the family, or known *BRCA1 *or *BRCA2 *mutation carriers [21]. After identifying these subjects we then collected comprehensive baseline epidemiologic, multi-generational pedigree and genetic data, and updated cancer and vital status through active ongoing follow-up from the eligible subjects and all available blood relatives who consented to join the BCFR. All individuals completed written informed consents and the overall study is approved by the Institutional Review Board at Columbia University Medical Center.

For this study, we further restricted eligibility to the 1,857 women from 938 families with at least one subsequent update on cancer and vital status, and who at cohort entry were aged 20 to 70 years and had no history of bilateral prophylactic mastectomy, or invasive or *in situ *breast cancer (women with both DCIS and LCIS were excluded). For this study, the family history information is defined based on each individual at the time they were recruited into the study. In this cohort of 1,857 women unaffected at baseline, 641 were without a first-degree female relative with breast cancer and only 110 were *BRCA1 *or *BRCA2 *positive. Thus, the cohort spans the continuum of risk including women at very high risk (mutation carriers) and those at lower risk (mutation negative and/or with more distant relatives with cancer). Figure 1 illustrates the range of remaining lifetime risk estimated from IBIS and BCRAT models.

### *BRCA1/2 *mutation testing

All self-identified Ashkenazi Jewish participants were screened for the three founder mutations, 185delAG and 5382insC in *BRCA1 *and 6174delT in *BRCA2*. In addition, for all non-Ashkenazi Jewish families, we screened the youngest affected individual using full sequencing methods. If the youngest affected individual had a mutation, the remaining family members were all tested for this family-specific mutation. In our cohort for this study, 800 women were tested for *BRCA *mutation, of which 110 tested positive. If the youngest affected member did not have a mutation in either *BRCA1 *or *BRCA2*, additional testing was not performed. For the purposes of our analyses, these women were separated and labeled 'not tested'. However, because the youngest affected member of these families did not have a mutation the probability that she had a mutation would be very low and thus for interpreation the women labeled 'not tested' can be interpreted as being 'negative' [27].

### Risk models

We assigned each subject a 10-year breast cancer risk using the software packages BCRAT and IBIS [3,6,9,10], using the models exactly as they can be applied in a clinical setting. The BCRAT model is based on a logistic regression model whose regression coefficients are combined with information on baseline age-specific hazard rates and competing mortality risks [3]. In the IBIS model the genetic risk is predicted assuming two autosomal dominant loci - *BRCA1/2 *- and a hypothetical low-penetrance dominant gene. Nongenetic risk factors are included via a proportional hazard model [6].

For BCRAT, we calculated 10-year risks using the latest update (August 2011) of the Statistical Analysis Systems (SAS) macro [28] that allows us to calculate absolute invasive breast cancer risks according to the BCRAT algorithm in batch mode. To check the reproducibility of our risk assignments, we compared the BCRAT risks assigned by the SAS macro to the weblink [20] for 10 randomly selected women. To assign 10-year IBIS risks, we used an external application provided by the orginal authors (personal communication). The results from the external application are exactly the same as those provided by the front line version available at the weblink [29] (IBIS risk evaluator - version 6.0.0).

### Statistical analysis

Women were classified into quartiles based on the predicted 10-year risk from the models. For each quartile, we used the survival data to estimate the 'observed' 10-year risk, defined as the probability *π *of developing breast cancer within 10 years of risk assessment and before dying of other causes. This probability is

(1) $$\pi =\int_{0}^{10}I_{B}\left(t\right)\text{exp}\left\{-\int_{0}^{t}\left[I_{B}\left(u\right)+I_{D}\left(u\right)\right]du\right\}dt,$$

where *I_B_(t) *and *I_D_(t) *denote the hazard rates for breast cancer and death, respectively, at *t *years from baseline [30]. If the death rate during the 10-year period is negligible and ignored, this probability reduces to $\pi =1-\text{exp}\left[-\int_{0}^{10}I_{B}\left(t\right)dt\right]-\int_{0}^{10}I_{B}\left(t\right)dt$, where the approximation holds since the breast cancer hazard rate is small. We used competing risk theory as outlined in [31] to estimate this probability in the presence of censoring due to incomplete 10-year follow-up. Specifically, we estimated *π *for each risk quartile by obtaining nonparametric estimates of the hazard rates *I_B_(t) *and *I_D_(t) *and using these estimates in equation (1). In the absence of censoring, the quartile-specific estimates $\hat{\pi }$would reduce to the number of subjects who developed breast cancer within 10 years of risk assignment divided by the quartile sample size of 1857/4 = 464.25.

We assessed model calibration by comparing the mean model-assigned risk to observed breast cancer incidence in each of the four assigned risk quartiles, using the Hosmer-Lemeshow (HL) chi-squared goodness-of-fit statistic [32]. To examine model performance across subgroups, we partitioned the cohort into covariate-specific subgroups and calculated observed risks, mean model-assigned risks, and the ratios of the two. Although the cohort contains pairs of first-degree relatives whose breast cancer risks may be correlated due to unmeasured genetic factors, we ignored this possible correlation in computing test statistics and confidence intervals (CIs), because the proportion of such pairs was small (less than one in a thousand pairs).

We assessed the models' abilities to discriminate the women who did and did not develop breast cancer within 10 years of risk assignment by estimating each model's area under the receiver-operator characteristic curve (AUC). This measure ranges from 0.5 (no discriminative ability) to 1 (perfect discrimination). We calculated AUC estimates using the R packages 'ROCR' and 'pROC' ignoring censored subjects, which is valid under the assumption that the censoring mechanism is unrelated to the risks of breast cancer and death [33]. We also calculated a case risk percentile (CRP) for each woman who developed breast cancer during the 10-year risk period (a case). A case's model-based CRP is the percentile of her assigned risk in the distribution of assigned risks of all women without the outcome (noncases). Larger CRPs for one model compared to another indicate better discrimination. (We calculated these CRPs ignoring censored subjects, which is valid under the assumption that the censoring mechanism is unrelated to the risks of breast cancer and death [33].) The mean CRP across all cases is the AUC [34]. We used the Wilcoxon signed rank to formally evaluate whether the median of the BCRAT CRP and the median of the IBIS CRPs are statistically significant for each subgroup that we compared. We also compared the two AUCs within different covariate-specific subgroups, to identify subgroups for whom one model outperforms the other. We used Statistical Analysis Systems (SAS™) software version 9.2 (SAS Institute, Chicago, IL, USA) to obtain two-tailed significance levels for the Wilcoxon signed rank test, and used the freely available software RMAP [35] to compute the calibration and descriptive statistics.

## Results

Table 1 presents characteristics of the 1,857 subjects who met the eligibility criteria. At baseline risk assignment, their median age was 44 years, 311 (17%) of them reported a prior breast biopsy, and 388 (21%) were Hispanic. In addition, 13% reported no female blood relative with breast cancer, 35% reported no first-degree female relative with breast cancer, and 75% reported no relatives with ovarian cancer. Among 800 subjects tested for *BRCA *mutations, 110 were positive, the remaining nontested are assumed negative (see Methods). Among all subjects, 83 developed breast cancer and 55 died of other causes within 10 years of baseline, 730 were breast-cancer-free 10 years after baseline and 989 were last observed without breast cancer within 10 years of baseline. The mean follow-up length was 8.1 years (range 0.1 to 14.5). A total of 76% of the cohort were observed for five or more years, and 4% were observed for one year or less.

**Table 1:** Distribution of risk factors in 1857 subjects from MNYR/New York site of the BCFR.

	Unaffected after 10 yrs	Follow-up <10 yrs	Died within 10 yrs	Breast cancer within 10 yrs	Total
**All subjects**	730	989	55	83	1857

**Age (yrs) at risk assignment**					
20-29	117	164	0	3	284
30-39	191	208	11	16	426
40-49	190	302	12	26	530
50-59	155	192	17	25	389
60-70	77	123	15	13	228

**Age (yrs) at first menarche**					
≥14	147	251	13	16	427
12-13	415	503	29	49	996
7-11	166	225	11	18	420
Unknown	2	10	2	0	14

**Age (yrs) at FLB**					
<20yr	27	118	8	2	155
20-24	161	227	15	18	421
25-29	148	187	10	18	363
≥30	117	145	10	20	292
Nulliparous	277	312	12	25	626

**Menopausal status**					
Pre	448	602	15	40	1105
Peri	67	104	4	6	181
Post	173	205	28	29	435
Unknown	42	78	8	8	136

**HRT use**					
Never	583	814	32	59	1488
Previous user (more than 5 yr ago)	12	18	2	0	32
Previous user (less than 5 yr ago)	31	69	6	10	116
Current user	104	88	15	14	221

**Number of breast biopsies**					
0	593	851	41	61	1546
1	98	101	7	16	222
≥2	39	37	7	6	89

**Atypical hyperplasia**					
Absent/Unknown	727	987	55	82	1851
Present	3	2	0	1	6

**First-degree female relatives with BC**					
0	251	336	28	26	641
1	397	544	22	40	1003
≥2	82	109	5	17	213

**All female relatives with BC**					
0	75	139	15	10	239
1	321	479	19	29	848
≥2	334	371	21	44	770

**Relatives with OC**					
0	555	752	27	61	1395
1	127	188	22	17	354
≥2	48	49	6	5	108

**BRCA mutation status**					
Total mutation positive	47	45	4	14	110
*BRCA1*	36	22	4	8	70
*BRCA2*	11	23	0	6	40

**Race/Ethnicity**					
Non-Hispanic White	651	509	36	74	1270
Hispanic	41	332	11	4	388
African American	6	42	5	0	53
Chinese	2	35	0	1	38
Filipino	0	1	0	0	1
Other Asian	4	20	2	1	27
Other	26	50	1	3	80

(*N *= 1857).

BC, breast cancer; FLB, first live birth; HRT, hormone replacement therapy; OC, ovarian cancer

### Overall assessment of BRCAT and IBIS models risks

**Figure 2:**
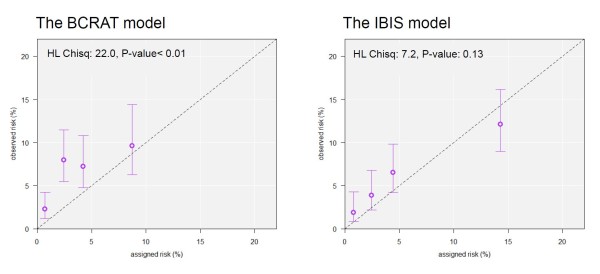
**Calibration of BCRAT and IBIS models**. The horzontal coordinates of points represent the mean 10-year assigned risks of BCRAT (left panel) and IBIS (right panel) within quartiles of assigned risk. Vertical coordinates represent quartile-specific estimates of 10-year breast cancer probabilities (observed risks). Vertical bars represent 95% confidence intervals for observed risks.

**Figure 3:**
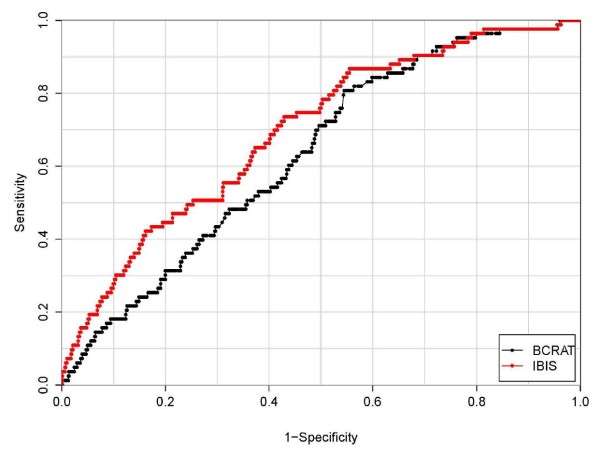
**Receiver operating characteristic (ROC) plots for BCRAT and IBIS assigned risks**. The area under the receiver operator characteristic curve (AUC) was 63.2% (confidence interval (CI) = 57.6% to 68.9%) for BCRAT and 69.5% (CI = 63.8% to 75.2%) for IBIS.

Figure 2 shows goodness-of-fit of BCRAT and IBIS assigned risks to the observed risks in the cohort. For BCRAT, the mean assigned risks were signficatly lower than the observed risks in the first three quartiles, and fit poorly overall (HL X_4_^2 ^= 22.0, *P *value <0.001). For IBIS, the mean assigned risks were nonsignificantly lower than the observed risks in quartiles 1, 2, 3 and nonsignificantly higher in quartile 4, with little evidence of poor fit (HL X_4_^2 ^= 7.2, *P *value = 0.13). The receiver operating characteristic **(**ROC) plots in Figure 3 indicate that IBIS also showed better discrimination between cases and noncases, with AUC of 69.5% (CI = 63.8 to 75.2%) compared to BCRAT AUC of 63.2% (CI = 57.6 to 68.9%). As seen in Figure 3, the assigned risk cutoff giving 80% specificity (corresponding to the value 0.20 on the horizontal axis) IBIS identified 44.6% of the cases, compared to a sensitivity of only 30.1% for BCRAT.

Although the cohort did not contain women with a history of *in situ *breast cancer, it did contain other women for whom BCRAT is not recommended, that is, women aged less than 35 years at risk assignment and women known to carry *BRCA *mutations. Accordingly, we also compared the two models after excluding these women from the cohort. We found that the superior performance of IBIS persisted: the HL statistic showed better calibration for IBIS (HL X_4_^2 ^= 6.3, *P *value = 0.18) than BCRAT (HL X_4_^2 ^= 12.7, *P *value = 0.01), and better discrimination (AUC = 63.7%, CI = 56.6 to 70.9%) than BCRAT (AUC = 57.5%, CI = 50.4 to 64.6%).

### Covariates associated with differential model performance

Which covariates unique to IBIS explain the observed differences in their performance? To address this question, we omitted information on second-degree family history from IBIS and found that the resulting 'pruned' risks, like those of BCRAT, were too low in the first three quartiles (HL X_4_^2 ^= 15.1, *P *value <0.01), and that the overall AUC estimate decreased to 67.8% (CI = 62.0 to 73.7). When *BRCA *status was also omitted from IBIS, the AUC decreased further to 62.2% (CI = 56.5 to 67.9), similar to that of BCRAT. Although the differences in these AUCs are not statistically significant, they suggest that, even when a risk model captures *BRCA *mutation status, its discrimination can be improved by including second-degree family history.

Table 2 gives observed and mean assigned risks and their ratios for subgroups defined by nongenetic and genetic covariates. The table shows that BCRAT risks are significantly lower than observed risks in almost all subgroups, including those containing women typically deemed at average risk (for example, those without a breast cancer family history and those not known to carry a *BRCA *mutation). The only exceptions were the subgroups of women with age at first birth less than 25 years, women with at least one prior breast biopsy, and women who are not Non-Hispanic White. Mean IBIS risks were significantly lower than observed risks in subgroups of women without first-degree relatives with breast cancer and without relatives with ovarian cancer, and were significantly higher than observed risks for mutation carriers.

**Table 2:** Ten-year breast cancer risks as observed and assigned by BCRAT and IBIS.

		Observed risk (%)		Assigned risk (%)		Observed/Assigned	
All subjects	N	Risk	CI	BCRAT	IBIS	BCRAT	IBIS
**Age (yrs) at risk assignment**						Ratio	Ratio
<35	**471**	1.91	0.91-3.98	0.58	2.11	3.29	0.91
35-49	**769**	7.03	5.06-9.68	2.96	6.13	2.38	1.15
50+	**617**	8.49	6.16-11.59	5.43	7.26	1.56	1.17

**Age (yrs) at first menarche**							
<14 yrs	**1416**	6.67	5.24-8.47	3.31	5.64	2.02	1.18
> = 14 or unknown	**441**	4.54	2.74-7.45	2.74	4.98	1.66	0.91

**Age (yrs) at FLB**							
<25 yrs	**576**	4.98	3.15-7.79	4.05	4.93	1.23	1.01
> = 25 or Nulliparous	**1281**	6.71	5.23-8.58	2.79	5.73	2.41	1.17

**Menopausal status**							
Pre	**1190**	5.13	3.77-6.94	2.04	4.51	2.51	1.14
Post	**667**	8.13	5.95-11.00	5.2	7.22	1.56	1.13

**HRT use**							
Never	**1488**	5.59	4.3-7.24	2.79	5.07	2.00	1.10
Ever	**369**	8.56	5.74-12.60	4.74	7.16	1.81	1.20

**Number of breast biopsies**							
0	**1546**	5.72	4.42-7.36	2.63	5.04	2.17	1.13
1+	**311**	8.7	5.72-13.02	5.87	7.72	1.48	1.13

**BMI (kg/m²)**							
<25	**1098**	5.80	4.36-7.67	2.98	5.25	1.95	1.05
> = 25	**759**	7.04	4.99-9.85	3.46	5.83	2.03	1.21

**First-degree female relatives with BC**							
0	**641**	5.84	3.93-8.61	1.67	3.43	3.50	1.70
1+	**1261**	6.48	4.98-8.38	3.97	6.57	1.63	0.99

**Second/third-degree relatives with BC**							
0	**969**	5.64	4.04-7.81	3.41	5.35	1.65	1.05
1+	**888**	6.83	5.10-9.09	2.92	5.64	2.34	1.21

**All relatives with OC**							
0	**1395**	6.09	4.72-7.84	3.26	4.53	1.87	1.34
1+	**463**	6.72	4.38-10.17	2.92	8.37	2.30	0.80

**BRCA mutation status**							
Test result positive	**110**	16.29	9.70-26.07	3.77	33.8	4.32	0.48
Test result negative	**690**	6.24	4.53-8.54	3.63	3.66	1.72	1.70
Untested	**1057**	5	3.46-7.17	2.82	3.73	1.77	1.34

**Race/Ethnicity**							
Non-Hispanic White	**1270**	7.09	5.65-8.85	3.52	6.15	2.01	1.15
Other	**587**	2.6	1.18-5.63	2.43	4.06	1.07	0.64

BC, breast cancer; FLB, first live-birth; HRT, hormone replacement therapy; OC, ovarian cancer.

**Figure 4:**
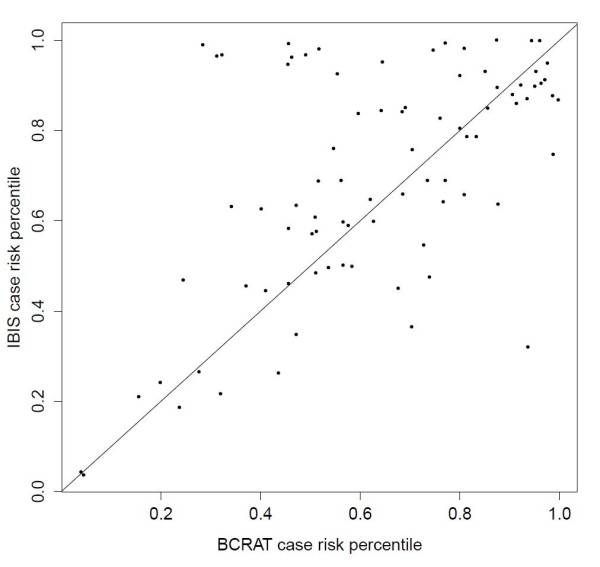
**Scatterplot of the case risk percentiles (CRPs)**. The horizontal and vertical coordinates of points give the BCRAT and IBIS CRPs, respectively, for the 83 breast cancer cases. Points above the diagonal line represent cases better identified by IBIS than BCRAT (since their IBIS risk percentiles are higher than their BCRAT risk percentiles). Points below the line correspond to cases better identified by BCRAT than IBIS. The mean CRP for a model is its area under the receiver-operator characteristic curve (AUC). A Wilcoxon signed-rank test of the 83 CRP pairs indicates that the IBIS AUC is significantly different than that of BCRAT (two-tailed *P *value = (0.04)).

As noted by Pepe and Longton [34], a useful measure of a model's ability to discriminate for individual breast cancer cases is provided by the percentile of her assigned risk in the distribution of assigned risks for all noncases, which we call her case risk percentile (CRP). Figure 4 shows a scatterplot of BCRAT and IBIS CRPs for the 83 women who developed breast cancer within 10 years of risk assignment. Points above the diagonal line (*N *= 46) represent cases whose subsequent breast cancer occurrence was better identified by IBIS than BCRAT, while points below the line (*N *= 37) represent cases better identified by BCRAT than IBIS. The mean CRP across cases for a model is its AUC. Using the Wilcoxon signed-rank test, we also found that the median IBIS CRP was statistically significantly different than that of BCRAT (two-tailed *P *value = 0.04). Figure 4 also illustrates that there are a number of outliers where the CRP for one model is substantially higher than for the other. For example, the three cases in the cluster in the upper left region of Figure 4 have appreciably larger IBIS CRPs than BCRAT CRPs. All three cases carry *BRCA *mutations, and one case has a first-degree relative with ovarian cancer, information used by IBIS but not BCRAT. In contrast, the outlying case in the lower right region with higher BCRAT than IBIS CRP had a prior biopsy, information used by BCRAT but not IBIS.

Comparison of BCRAT and IBIS AUCs within covariate-specific subgroups is provided in Table 3. Statistically significant differences are shown in boldface. IBIS shows better discrimination than BRCAT in all but one of the 13 comparisons showing statistically significant differences; the exception was the subgroup of women with at least one prior biopsy, where the BCRAT CRP was significantly higher than that of IBIS.

**Table 3:** Mean case risk percentiles for BCRAT and IBIS among subgroups of subjects.

	N	BCRAT	IBIS	*P *value^ª^
**Age (yrs) at risk assignment**				
<35	7	18.8	31.2	0.2969
35-49	38	56.9	66.9	**0.0377**
50+	38	77.7	79.1	0.7489

**Age (yrs) at first menarche**				
<14 yrs	67	63.6	69.7	**0.0717**
> = 14 or unknown	16	61.2	68.6	0.4637

**Age (yrs) at FLB**				
<25 yrs	20	67.7	61.3	0.2268
> = 25 or nulliparous	63	61.8	72.1	**0.0022**

**Menopausal status**				
Pre	43	51.8	61.9	**0.0196**
Post	40	75.4	77.6	0.6064

**HRT use**				
Never	59	58.3	66.8	**0.0149**
Ever	24	75.1	76.1	0.9119

**Number of breast biopsies**				
0	61	56.4	68.7	**0.0001**
1+	22	81.9	71.7	**0.0118**

**BMI (kg/m²)**				
<25	48	62.5	65.6	0.9919
> = 25	35	64.1	74.8	**0.0014**

**First-degree female relatives with BC**				
0	26	45.1	56.8	**0.0967**
1+	57	71.4	75.2	0.1816

**Second/third-degree relatives with BC**				
0	37	67.4	70.7	0.7617
1+	46	59.8	68.5	**0.0185**

**Relatives with OC**				
0	61	65.4	67.5	0.5412
1+	22	57.0	75.1	**0.0094**

**BRCA mutation status**				
Test result positive	14	61.4	98.1	**0.0001**
Test result negative	37	68.8	65.5	0.1457
Untested	32	57.5	61.6	0.1823

**Ethnicity**				
Non-Hispanic White	74	65.2	72.1	**0.0638**
Other	9	46.6	48.1	0.4961

ªUsing Wilcoxon signed rank test. BC, breast cancer; FLB, first live birth; HRT, hormone replacement therapy; OC, ovarian cancer.

## Discussion

Breast cancer risks for women in the present cohort span the continuum of risk. Our intention was to compare two commonly used models that include nongenetic factors, BCRAT and IBIS, as used by clinicians. We observed better overall calibration and discrimination for IBIS than for BCRAT, in agreement with the findings of Amir *et al *[16], with the latter study spanning a narrower range of risks. The higher performance of IBIS persisted when we exluded women for whom BCRAT is not recommended. We also found better performance for IBIS in almost all covariate-specific subgroups, except for Hispanic and nonwhite women, and women with a prior breast biopsy. Race is an important predictor of breast cancer risk [36], and hereditary patterns and mutation prevalences differ by race and ethnicity [37]. The BCRAT model was updated, in 2008, to incorporate revised estimates for African American women [8] and in 2011, to include projections for Asian and Pacific Islander Americans. The BCRAT risks were lower than observed risks for almost all other subgroups, most notably those for whom the model is not recommended: *BRCA1/2 *mutation carriers and women under age 35 years at risk assignment. Overall, our cohort is of higher risk than the general U.S. population. For example, compared to SEER rates based on the age distribution of our cohort, our observed rates were 3.1 times that expected of an average risk population. Thus, while the overall improved performance of IBIS over BCRAT may not be unexpected given our higher risk cohort, what was unexpected was that this improved performance extended to subgroups containing woman considered at average risk, such as those with no family history and no *BRCA1/2 *mutations.

By comparing the performance of the full IBIS model to a pruned version lacking second-degree family history information, we found that this extended family information increased the AUC estimate, despite the pruned model's capture of *BRCA *mutation status. This improvement, although not statistically significant, is nevertheless plausible, as extended family history captures all of the many breast cancer genetic risk factors in addition to *BRCA1/2 *mutations as well as nongenetic shared familiar environmental factors not captured in the model. A practical barrier to the broad use of models incorporating extended family history data are patients' incomplete knowledge about the health of their more distant blood relatives.

Risks assigned by the IBIS model also discriminated future cases from noncases better than did those assigned by BCRAT, although the differences were not statistically significant. Discrimination was better for IBIS than BCRAT in almost all subgroups, and as expected, the difference was particularly large for *BRCA1/2 *mutation carriers. Only in women with a prior breast biospy was the discrimination better in BCRAT. BCRAT includes number of biopsies, regardless of their outcome, while the IBIS model only includes atypical hyperplasia diagnosed via biopsy. This may limit the precision of IBIS estimates, because pathology of biopsies is incompletely obtained from self-report, while number of biopsies can be more readily recalled and accurately reported.

Oncologists and genetic counselors would be well served by a single model that avoids having to choose among several models on this basis of patient characteristics. Our cohort spans the continuum of risk with a proportion of the women below population average risk (see Figure 1); this wide variation in individual risk makes it an interesting population with which to examine model performance across subgroups of women. Further research is needed to develop and validate a model that does well for all women.

Enhancing the IBIS model with additional risk factors, such as number of breast biopsies and race/ethnicity, may further improve its performance. In addition, the IBIS model would need expansion to handle the risks of women with atypical hyperplasia, since the model is not recommended for these women due to its poor discriminiation among them (AUC 0.54) [38].

## Conclusions

In summary, we found that the IBIS model performed better in this cohort whose risks span the continuum of breast cancer risk. This was true even in subgroups containing women typically considered average risk (for example, no family history of breast cancer, *BRCA1/2 *mutation negative). Interestingly, the highest quartile of BCRAT-assigned risks was the only one in which the mean BCRAT risk did not differ signficantly from the observed risk. Thus, not only did IBIS outperform BCRAT in subgroups whose risks are typically considered average (the patients for whom BCRAT is used clinically) but the BCRAT model was well calibrated only in the risk group in which it is unlikely to be used by clinicians, that is, the highest quartile of assigned risk. These findings need replication in other large cohorts spanning a broad range of risks. They suggest the complexity of applying risk models in the clinic based on *a priori *assumptions of risk defined by family history and genetic status. Models that have been developed based on extended family history and genetic data, such as the IBIS model, may perform well in women considered at average risk. Extending models that already capture extended family history and genetic information to include a larger array of nongenetic risk factors may help risk models play a major role in disease prevention.

## Abbreviations

AUC: area under the receiver-operator characteristic curve; BCFR: Breast Cancer Family Registry; BCRAT: Breast Cancer Risk Assessment Tool; CI: confidence interval; CRP: case risk percentile; HL: Hosmer-Lemeshow chi-squared goodness-of-fit test; IBIS: International Breast Cancer Intervention Study.

## Competing interests

The authors declare that they have no competing interests

## Authors' contributions

MBT is the PI of the parent study and initiated this current study. ASQ, ASW, MBT worked closely together at all stages of this current study and drafted the manuscript. ASQ carried out the analysis. ASW developed the methods for quantile-specific risk performance metrics and led the overall statistical analyses. TS was integral to the refinement of the family pedigree data and other data used in the risk models. ASQ, ASW, TS, KS and MBT contributed to the interpretation of the data, revised the manuscript, and approved of the final manuscript.
